# n-Butyl Benzyl Phthalate Promotes Breast Cancer Progression by Inducing Expression of Lymphoid Enhancer Factor 1

**DOI:** 10.1371/journal.pone.0042750

**Published:** 2012-08-08

**Authors:** Tsung-Hua Hsieh, Cheng-Fang Tsai, Chia-Yi Hsu, Po-Lin Kuo, Edward Hsi, Jau-Ling Suen, Chih-Hsing Hung, Jau-Nan Lee, Chee-Yin Chai, Shao-Chun Wang, Eing-Mei Tsai

**Affiliations:** 1 Graduate Institute of Medicine, Kaohsiung Medical University, Kaohsiung, Taiwan; 2 Institute of Clinical Medicine, College of Medicine, Kaohsiung Medical University, Kaohsiung, Taiwan; 3 Center of Excellence for Environmental Medicine, Kaohsiung Medical University, Kaohsiung, Taiwan; 4 Department of Pediatrics, Kaohsiung Medical University Hospital, Kaohsiung Medical University, Kaohsiung, Taiwan; 5 Department of Pathology, Kaohsiung Medical University Hospital, Kaohsiung Medical University, Kaohsiung, Taiwan; 6 Department of Cancer and Cell Biology, University of Cincinnati College of Medicine, Cincinnati, Ohio, United States of America; 7 Department of Obstetrics and Gynecology, Kaohsiung Medical University Hospital, Kaohsiung Medical University, Kaohsiung, Taiwan; Sun Yat-sen University Medical School, China

## Abstract

Environmental hormones play important roles in regulating the expression of genes involved in cell proliferation, drug resistance, and breast cancer risk; however, their precise role in human breast cancer cells during cancer progression remains unclear. To elucidate the effect of the most widely used industrial phthalate, n-butyl benzyl phthalate (BBP), on cancer progression, we evaluated the results of BBP treatment using a whole human genome cDNA microarray and MetaCore software and selected candidate genes whose expression was changed by more than ten-fold by BBP compared with controls to analyze the signaling pathways in human breast cancer initiating cells (R2d). A total of 473 genes were upregulated, and 468 were downregulated. Most of these genes are involved in proliferation, epithelial-mesenchymal transition, and angiogenesis signaling. BBP induced the viability, invasion and migration, and tube formation in vitro, and Matrigel plug angiogenesis in vivo of R2d and MCF-7. Furthermore, the viability and invasion and migration of these cell lines following BBP treatment was reduced by transfection with a small interfering RNA targeting the mRNA for lymphoid enhancer-binding factor 1; notably, the altered expression of this gene consistently differentiated tumors expressing genes involved in proliferation, epithelial-mesenchymal transition, and angiogenesis. These findings contribute to our understanding of the molecular impact of the environmental hormone BBP and suggest possible strategies for preventing and treating human breast cancer.

## Introduction

Phthalates, a group of environmental hormones, disrupt endocrine function and affect human health by mimicking the function or inhibiting the action of steroid receptors. They are present in cosmetics, medical products, and toys and can be absorbed through the skin [1] or ingested if food comes into contact with plastic film wrappers [2]. Previous studies have demonstrated that phthalates are associated with an increased risk of breast cancer [3], and the association between phthalate exposure and glutathione S-transferase M1 polymorphism in adenomyosis [4]. Reproductive evidence has shown that phthalates have an adverse effect on androgenic action in males and alter estradiol synthesis in females [5], [6].

Phthalate esters like n-butyl benzyl phthalate (BBP) are widely used plasticizers that have weak estrogenic activity and compete with estradiol for binding to estrogen receptor (ER) [5]. Several studies have revealed that 10 µM BBP increases the proliferation ability and induces mitosis in the ER-positive breast cancer cell lines MCF-7 and ZR-75 [7], [8]. Furthermore, BBP can induce expression of the oncogenes *c-Myc* and *HDAC6* (encoding histone deacetylase 6) in ER-negative breast cancer cell lines [9]. BBP promotes activation of breast cancer and may reduce clinical effectiveness of chemotherapy. The investigation examined whether there is a relationship between BBP and tamoxifen chemotherapy, finding that BBP promotes resistance to tamoxifen by inhibiting tamoxifen-induced apoptosis in breast cancer cells [10]. However, whether BBP exposure affects the breast cancer initiating cell system is largely unknown.

We previously reported the generation of the first estrogen-responding tumor-initiating cell line termed R2d cells [11] which were isolated from human breast epithelial cells (HBECs) [12]. R2d cells harbor stem cell characteristics in self renewal and pluripotent differentiation potential, express stem cell markers (CD44^+^/CD24^−^), and are ER-positive. Importantly, in response to estrogen stimulation R2d cells gain cell growth activity, undergo epithelial-mesenchymal transition, promote angiogenesis, and initiate tumor development [11]. Thus, R2d is a unique cancer initiating cells possessing estrogen-dependent tumorigenic potential.

To understand the global impact of BBP on gene expression in R2d cells, we used whole-genome screening with a high-density microarray assay and analyzed the network pathway. Moreover, we examined the impact of BBP on biological function and unveiled significant candidate genes. We show that BBP led to upregulation of lymphoid enhancer-binding factor 1 (LEF-1) and promoted cell progression in R2d cells.

## Results

### BBP increases the viability of R2d cell lines

**Figure 1:**
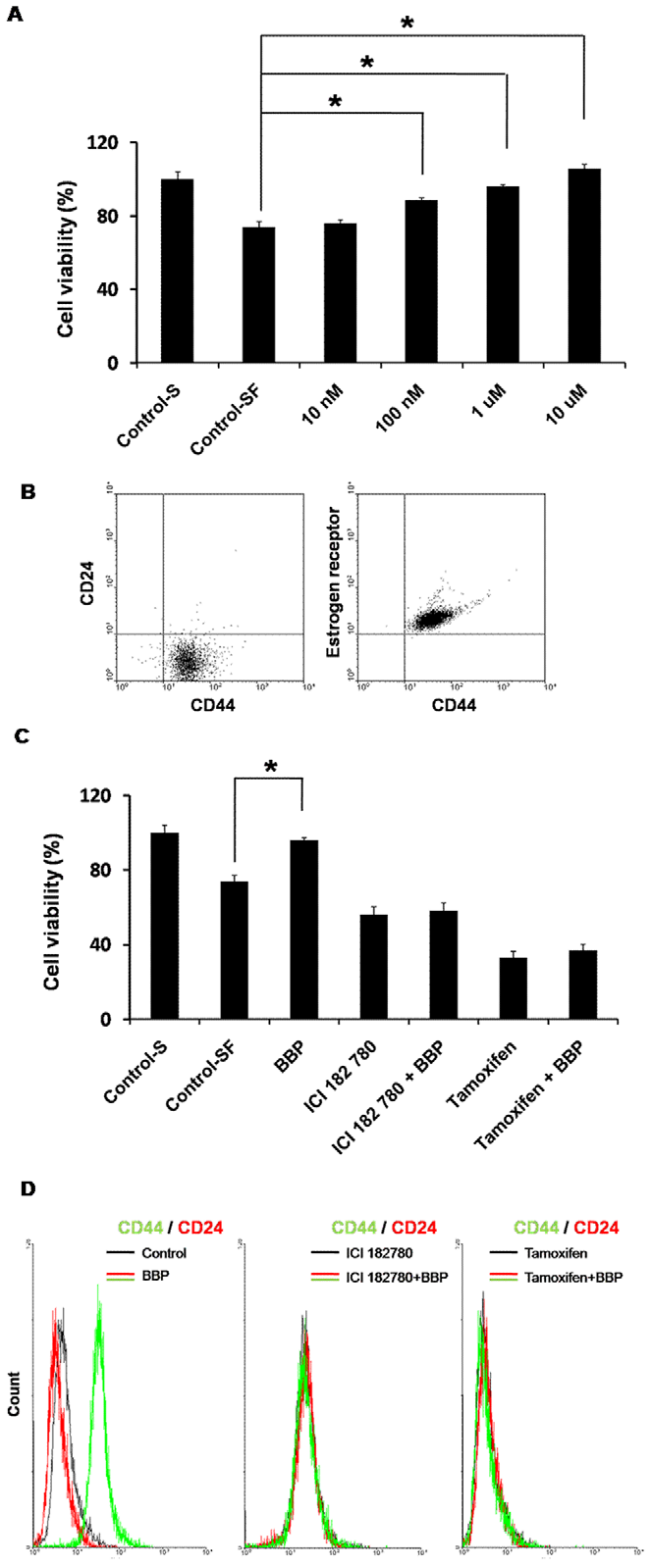
Effects of BBP on the viability of cultured R2d cells. (A) R2d cells were exposed to 10 nM, 100 nM, 1 µM, or 10 µM BBP in serum-free medium for 24 h. Cell viability was determined with the XTT assay. (B) CD44, CD24 surface markers and estrogen receptor was stained by flow cytometry in R2d. (C,D) R2d cells were pretreated with ICI182780 (1 uM), tamoxifen (1 uM) for 1 h, and then treated with 1 µM of BBP for an additional 24 h. Viability of cells and CD44 was measured. These data represent the mean ± SE of three experiments. Each asterisk denotes a significant difference compared to the control (P<0.05; one-tailed Student's t-test). Control-S: control cells grown in serum containing media, Control-SF: control cells grown in serum-free media.

To investigate the estrogenic effects of BPP on cultured R2d cell, we first used XTT assay to examine cell viability following exposure to different concentration of BBP for 24 h. The effects of BBP on cell viability were analyzed with a 3-(1-(phenylaminocarbony)-3,4-tetrazolium)-bis-(4-methoxy-6-nitro)-benzene sulfonic acid hydrate (XTT) assay in breast cancer cells. R2d cells were stimulated with different concentrations of BBP (10 nM, 100 nM, 1 µM, and 10 µM) for 24 h, and the wavelength was measured with an ELISA reader. As shown in Figure 1A, cell viability significantly increased in a dose-dependent manner, with changes of 1.20-fold at 100 nM, 1.30-fold at 1 µM, and 1.43-fold at 10 µM. Furthermore, we compared CD44/CD24/ER expression in R2d cells. The results show that the majority of R2d cells sorted into the CD44^+^/CD24^−^/ER^+^ fraction (Fig. 1B). To further examine the estrogenic effects of BBP in R2d cells, the estrogen inhibitors ICI182780 (1 uM) and tamoxifen (1 uM) was used to analyze the cell viability and tumorigenicity surface markers CD44 expression. The results show that ICI182780 and tamoxifen inhibit BBP-induced viability (Fig. 1C) and CD44 expression (Fig. 1D) in R2d cells. These data demonstrate that BBP, which can mimic estrogen to promote viability, tumorigenicity and state the estrogenic effects of BBP in R2d cells.

### cDNA microarray and signaling pathways

**Figure 2:**
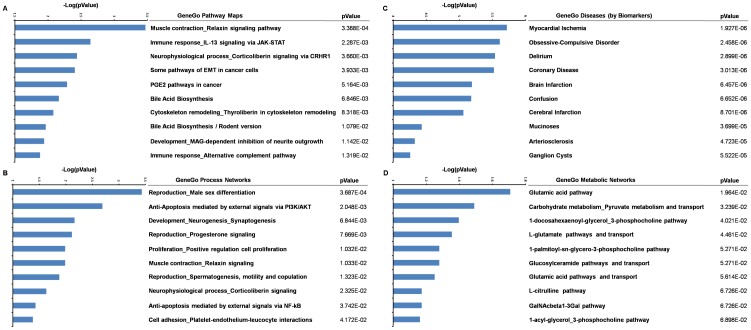
Pathway analysis of genes whose expression was significantly altered by BBP treatment compared with control. The top ten GeneGO pathway maps (A), cellular processes (B), diseases (C), and metabolic networks (D) in BBP-treated cells are shown. These gene pathways were analyzed with MetaCore software.

To examine the role of BBP in breast cancer, we used a whole-genome cDNA microarray to screen BBP regulation of gene expression in R2d cells. We added 1 µM BBP to R2d cells for 24 h before isolating the total RNA and synthesized cDNA. The microarrays were detected with Illumina Inc. BeadStudio version 3.3.7 system and analyzed with MetaCore software. Microarray analysis showed that the expression of 941 genes was changed more than ten-fold following BBP treatment compared with the control; 473 were upregulated, and 468 were downregulated. The top 30 upregulated genes are shown in Table 1. To further evaluate the functional pathways of genes up- or downregulated more than ten-fold, we analyzed Pathway Maps, Cellular Processes, Diseases, and Metabolic Networks using MetaCore software. We found that the genes expressed at a higher level were related to the immune response (P = 0.002287), the epithelial-to-mesenchymal transition (EMT; P = 0.003933), the PGE2 pathway (P = 0.005164), and cytoskeleton remodeling (P = 0.008318) in the top ten GeneGo Pathway Maps (Fig. 2A) and neurogenesis/synaptogenesis (P = 0.006844), positive regulation and cell proliferation (P = 0.01032), and cell adhesion (P = 0.04172) in the top ten GeneGo Cellular Process Networks(Fig. 2B). Overall, the software analysis (Fig. 2) provides that BBP may promote cell proliferation, EMT, and angiogenesis in R2d cells.

**Table 1:** Top 30 genes upregulated by BBP treatment compared with the control.

No.	Symbol	Fold change	Gene description
1	LACCI	328.3826	laccase (multicopper oxidoreductase) domain containing 1
2	GSTZ1	245.4594	glutathione transferase zeta 1
3	FNBP1L	243.0455	formin binding protein 1-like
4	FAM150A	151.7533	family with sequence similarity 150, member A
5	SRD5A2	142.7481	steroid-5-alpha-reductase, alpha polypeptide 2
6	GDF11	136.7424	growth differentiation factor 11
7	ZNF610	85.44753	zinc finger protein 610
8	ZFHX2	85.3102	zinc finger homeobox 2
9	HAVCR2	77.37178	hepatitis A virus cellular receptor 2
10	PCDHGA4	75.05667	protocadherin gamma subfamily A, 4
11	KIAA1377	72.16377	
12	TPK1	68.9534	thiamin pyrophosphokinase 1
13	POU4F2	65.87636	POU class 4 homeobox 2
14	LGR8	61.58894	relaxin/insulin-like family peptide receptor 2
15	LASS3	60.88636	ceramide synthase 3
16	CR1	58.53471	complement component (3b/4b) receptor 1
17	KRTHB6	53.54553	keratin 86
18	CYP4Z1	52.63741	cytochrome P450, family 4, subfamily Z, polypeptide 1
19	TNFRSF11B	50.60729	tumor necrosis factor receptor superfamily, member 11b
20	C1ORF84	50.29234	chromosome 1 open reading frame 84
21	PER2	49.22897	period homolog 2 (Drosophila)
22	ESRRB	48.91599	estrogen-related receptor beta
23	SEC24A	47.20217	SEC24 family, member A (S. cerevisiae)
24.	OR7G2	46.38392	olfactory receptor, family 7, subfamily G, member 2
25	KU-MEL-3	40.18513	
26	PCSK2	39.77561	proprotein convertase subtilisin/kexin type 2
27	LACE1	38.6673	lactation elevated 1
28	C9ORF27	38.47661	long intergenic non-protein coding RNA 474
29	ZNF683	38.20434	zinc finger protein 683
30	SPACA5	38.15936	sperm acrosome associated 5

### BBP increases the in vitro viability, invasion and migration of R2d cells and in vivo angiogenesis of breast tumors

**Figure 3:**
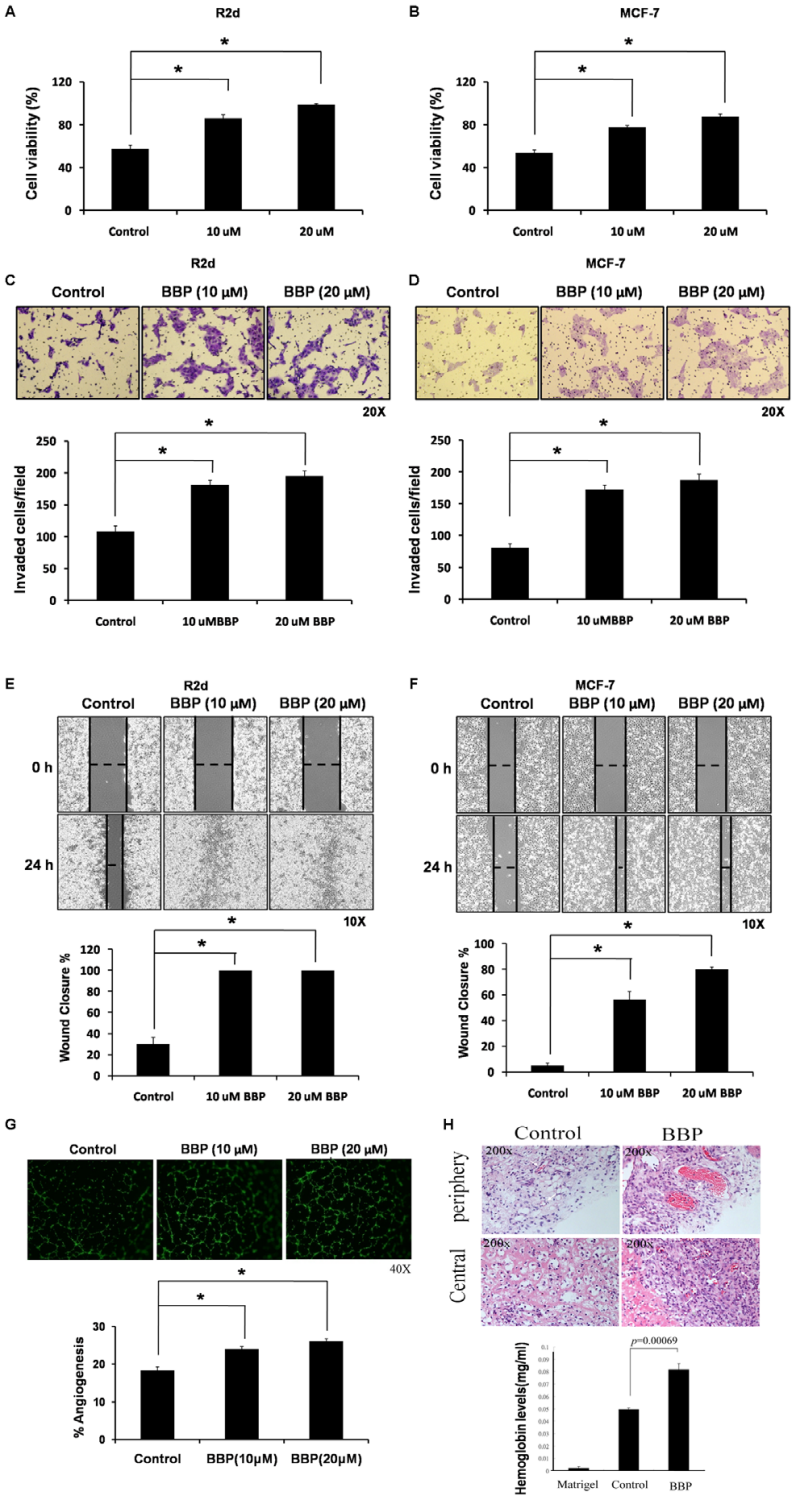
BBP-mediated cell viability, invasion and migration of breast cancer cells and angiogenesis in breast cancer. Breast cancer cells were exposed to 10 or 20 µm BBP for 24 h. The viability of R2d (A) and MCF-7 (B) cells was analyzed with the XTT assay. Invasion and migration were assessed with invasion assays (C, D) and the wound healing assay (E, F). The horizontal dashed line in panels E and F indicates the width of the wound. For the in vitro angiogenesis study, HUVECs were seeded into BioCoated plates, and added the medium that was obtained from the MCF-7 replacing medium with or without BBP (10, 20 µM) for 18 h. The capillary-like structures formed by HUVECs were stained with Calcein-AM and imaged by fluorescence microscopy. The tube formation number in different concentrations of HUVECs was calculated by MetaMorph software (Molecular Devices) (G). For the in vivo angiogenesis study, MCF-7 cells were mixed with phenol red–free Matrigel and injected into the flanks of nude mice for 15 days. In vivo angiogenesis was determined by the levels of red blood cells in tissue sections containing Matrigel plugs (H, top). The hemoglobin levels were determined with the Drabkin method (H, bottom). The data represent the mean ± SE of three experiments. Each asterisk denotes a significant difference compared to the control (P<0.05; one-tailed Student's t-test).

To confirm the functional pathway identified by MetaCore analysis and determine whether BBP similar to estradiol have the estrogenic effects for mediating tumorigenicity of R2d by inducing in vitro cell proliferation, invasion, migration and in vivo angiogenesis. Furthermore, we examined the effect of BBP on tumorigenicity in various types of cell lines. R2d and MCF7 breast cancer cell lines, containing side population cells with cancer initiating characteristics [12], [13], were treated with BBP to analyze the biological functions. We first confirmed the effects of BBP (10 µM and 20 µM) on cell viability with the XTT assay. BBP increased the viability of R2d cells (10 µM, 1.49-fold; 20 µM, 1.72-fold) (Fig. 3A) and MCF-7 cells (10 µM, 1.44-fold; 20 µM, 1.63-fold) (Fig. 3B). Furthermore, invasion assays (Fig. 3C, D) and wound healing assays (Fig. 3E, F) showed that cell invasion and migration were enhanced by treatment with BBP for 24 h. We next investigated whether BBP increases angiogenesis in breast cancer cells. The tube formation ability of human umbilical vein endothelial cells (HUVECs) and a Matrigel plug was used to analyze the angiogenesis ability in vitro and in vivo, respectively. As shown in Fig. 3G, the in vitro tube formation of HUVECs significantly increased in a dose-dependent manner in the presence of BBP. Furthermore, staining of tissue sections with hematoxylin and eosin showed that BBP increased the production of functional blood vessels (Fig. 3H, Top) and induced hemoglobin expression in the Matrigel plug (Fig. 3F, Bottom, P = 0.00069). These results suggest that BBP treatment increased breast cancer cell viability, invasion and migration in vitro, and increased angiogenesis in vivo.

### Functional description of BBP-regulated genes in human breast cancer

To further assess the biological processes and molecular functions of the BBP-regulated genes, we selected three functional groups regarding cancer progression from the Pathway Maps and Cellular Process pathways. These functional groups included proliferation-positive regulation of cell proliferation (proliferation pathway), some pathways of EMT in cancer cells (EMT pathway), and PGE2 pathways in cancer (angiogenesis pathway [14]–[16]). Their characteristics were similar to BBP mediating the biological function in breast cancer. The average fold changes of selected genes in these functional groups are listed in Table 2. Eight genes involved in the proliferation pathway group from the GeneGo Cellular Process pathway (CRIPTO, FGF, EDNRA, LMO1, VEGF-D, Galpha(i)-specific peptide GPCRs, PKA-reg (cAMP-dependent), and LEF-1) were activated by BBP. Furthermore, genes from the EMT pathway group (PDGF receptor, PDGF-R-alpha, EDNRA, and LEF-1) and angiogenesis pathway group (LEF-1, ADCV, TCF (LEF-1), and PKA-reg (cAMP-dependent)) were also induced by BBP. A total of 11 significantly induced genes were selected for further analysis.

**Table 2:** Functional description of the candidate genes whose expression was altered by BBP treatment in R2d cells.

Functional group	Symbol	Gene description	Fold change
**Proliferation_Positive regulation cell proliferation**			
	CRIPTO	teratocarcinoma-derived growth factor 1	58.53471
	FGF	fibroblast growth factor	31.15184
	EDNRA	endothelin receptor type A	10.83108
	LMO1	LIM domain only 1 (rhombotin 1)	11.10314
	VEGF-D	c-fos induced growth factor (vascular endothelial growth factor D)	14.86143
	Galpha(i)-specific peptide GPCRs	guanine nucleotide binding protein (G protein), q polypeptide	17.48679
	PKA-reg (cAMP-dependent)	protein kinase, cAMP-dependent, regulatory, type I, beta	25.95046
	LEF-1	lymphoid enhancer-binding factor 1	29.59359
**Some pathways of EMT in cancer cells**			
	PDGF receptor	platelet-derived growth factor receptor	36.39098
	PDGF-R-alpha	Alpha platelet-derived growth factor receptor precursor	50.03915
	EDNRA	endothelin receptor type A	10.83108
	LEF-1	lymphoid enhancer-binding factor 1	29.59359
**PGE2 pathways in cancer**			
	LEF-1	lymphoid enhancer-binding factor 1	29.59359
	ADCV	Adenylate cyclase	38.15936
	TCF(LEF-1)	transcription factor, T-cell specific	18.74659
	PKA-reg (cAMP-dependent)	protein kinase, cAMP-dependent, regulatory, type I, beta	25.95046

**Figure 4:**
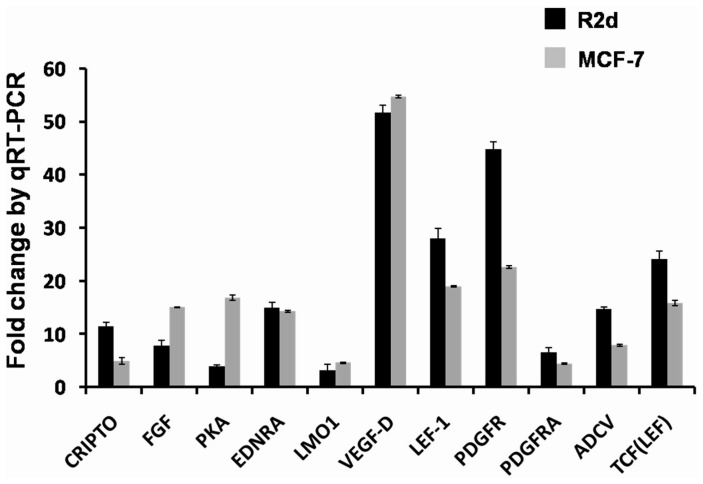
The effect of BBP on expression of selected genes in breast cancer cells. The expression of CRITPO, FGF, PKA, EDNRA, LMO1, VEGF-D, LEF-1, PDGFR, PDGFRA, ADCV, and TCF (LEF) was examined in R2d and MCF-7 cells treated with 1 µM BBP using qRT-PCR. The data represent the mean ± SE of three experiments.

To confirm the reliability of the fold changes identified with microarray analysis, the differentially expressed genes were examined with qRT-PCR. We used R2d and MCF-7 cells to characterize gene expression in response to BBP (1 µM) after 24 h. As shown in Figure 4, all examined genes were upregulated by BBP treatment, with VEGF-D, PDGFR, and LEF-1 being the three most highly expressed genes in both R2d and MCF-7 cells.

### LEF-1 is required for BBP-mediated cell viability, invasion and migration

**Figure 5:**
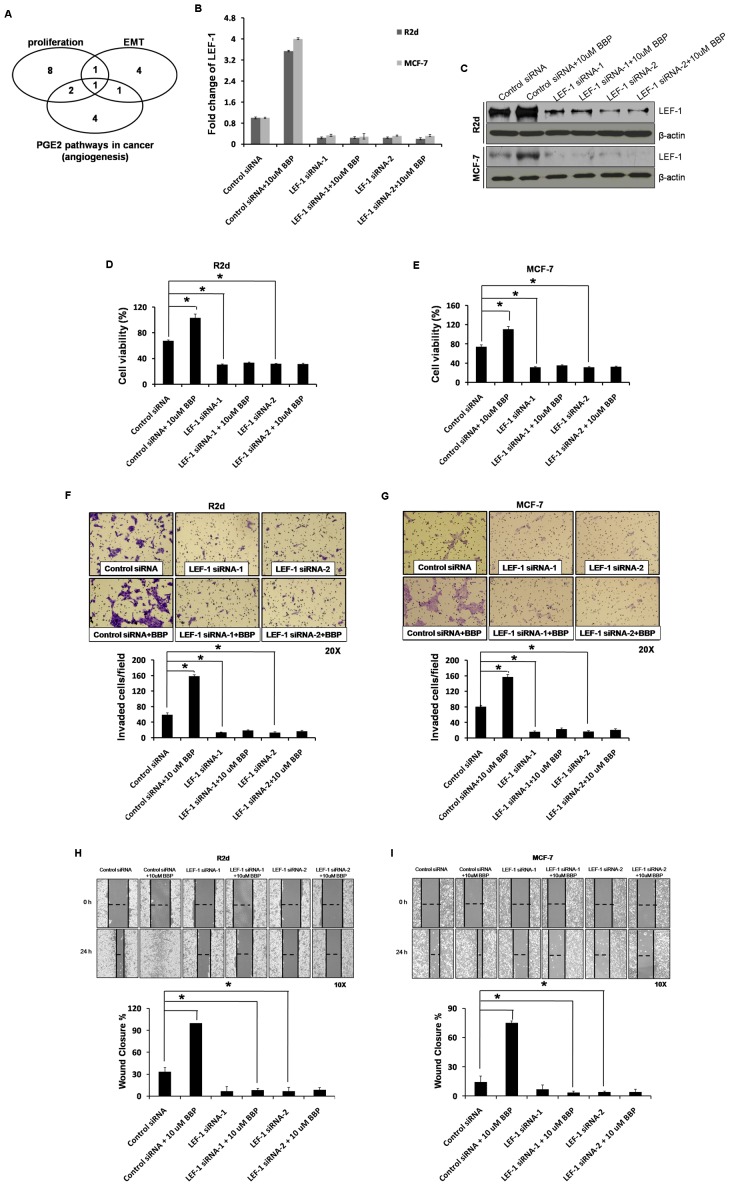
Role of LEF-1 in breast cancer cells treated with BBP. (A) One identified gene, LEF-1, was found to be in the proliferation pathway group, the EMT pathway group, and the angiogenesis pathway group. (B, C) Silencing of LEF-1 was performed by transfecting LEF-1 siRNA-1 (5 nM) or LEF-1 siRNA-2 (5 nM), and LEF-1 expression was detected with qRT-PCR, western blot and compared to cells transfected with control siRNA. (D, E) Breast cancer cells were pretransfected with control siRNA or siRNA for LEF-1 (siRNA-1/2) and then treated with BBP for 24 h. Cell viability was analyzed with the XXT assay in R2d (D) and MCF-7 (E) cells. Cells treated as in panels D and E were assessed with the invasion assay (F, G) and wound healing assay (H, I). The horizontal dashed line in panels H and I indicates the width of the wound. The data represent the mean ± SE of three experiments. Each asterisk denotes a significant difference compared to the control (P<0.05; one-tailed Student's t-test).

Further assessment of the results acquired from the microarray assays showed that LEF-1 gene was consistently identified in the proliferation pathway group, the EMT pathway group, and the angiogenesis pathway group (Fig. 5A). The expression of this gene, LEF-1, was 29.6-fold and 28.0-fold higher in BBP-treated cells, respectively, in microarray and qRT-PCR analysis.

To examine the role of LEF-1, we suppressed LEF-1 using siRNA and examined viability and invasion following BBP treatment; hence, LEF-1 was silenced with specific siRNAs in both R2d and MCF-7 cells (Fig. 5B, C). The down-regulation of LEF-1 cells were treated with 10 µM BBP for 24 h and analyzed for cell viability, invasion and migration. LEF-1 silencing blocked BBP from activating cell growth (Fig. 5D, E), invasion (Fig. 5F, G) and migration (Fig. 5H, I). These data suggest that BBP mediates cell viability, invasion and migration via LEF-1 in breast cancer.

## Discussion

**Figure 6:**
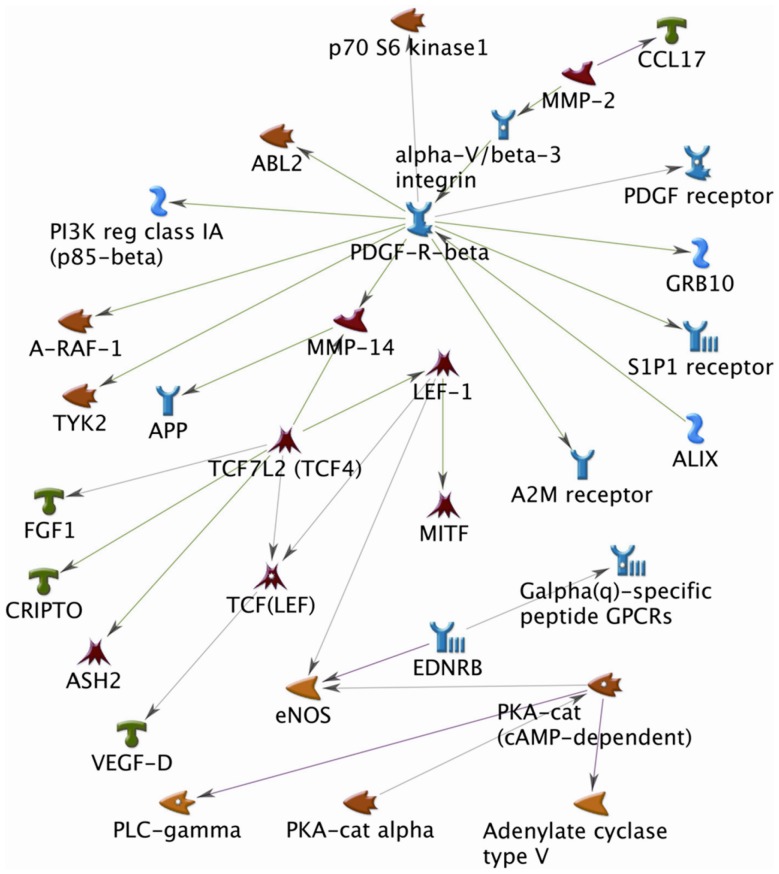
The network pathway associated with the proteins differentially expressed in response to BBP treatment. See text for details.

Breast cancer progression includes the maintenance of proliferation [17], metastasis [18], and angiogenesis [19]. The effects of estradiol on progression in breast cancer cells have been widely studied, but relatively little information is known about the impact of environmental hormones to breast cancer development. In this study, we approach this fundamental question by understanding the cellular effects of BBP in the transforming phenotypes of R2d cells. In current study, we used a high-density microarray to identify genes whose expression levels were significantly altered by BBP and to select candidate genes from three functional groups including proliferation, EMT, and angiogenesis. These candidate genes for a response to BBP are shown schematically in a relationship pathway in Figure 6. Among the genes in these functional groups, CRITPO, FGF, PKA-cat, VEGF-D, and PDGF-R-alpha are well-known mediators of cancer progression [20]–[24], and overexpression of FGF, VEGF-D, and PDGF-R-alpha is negatively correlated with patient survival [21], [25]. Moreover, the VEGF-D and PKA signaling pathway is associated with environmental hormones and mediates cancer cell proliferation and invasion [9], [26].

R2d cells have the characteristic of CD44 and Oct-4-positive [27], and its parent, M13SV1R2 has the ability to develop tumor [28], self-renewal, resistance to drugs and apoptosis [29]. Moreover, R2d maintaining the stem cells characteristics could be a novel eligible material for analyzing the estrogenic response. Our early study also showed that BBP enhanced epithelial-to-mesenchymal transition (EMT) [30] which was recently demonstrated to exhibit enhanced cancer-initiating cell features [31]–[33] in R2d cell lines. Thus, it is reasonable to refer the R2d cells as cancer initiating cells which can be promoted by hormonal stimulation. On the other hand, it should be pointed out that emerging evidence shows that breast cancer progenitor cells or stem cells can be derived from an estrogen receptor (ER)-positive niche. Thus, the estrogen-dependent nature of tumorigenicity of R2d cells does not preclude their function in cancer initiation.

ER negativity in cancer stem cells is supported by several studies [34], [35]. However, other studies have also shown that CD44^+^/CD24^−^ cancer stem cells can evolve from ER-positive cells, and that estrogen induces expansion and tumor initiation activity of cancer stem cells [36]–[40]. Our finding corroborates these reports in that BBP, like estrogen, stimulates proliferation, invasiveness, and angiogenesis potential of R2d cells. More importantly, BBP treatment expands CD44^+^/CD24^−^ population of R2d cells, which is blocked by the estrogen antagonists ICI182,780 and tamoxifen [5], [36]. It is also noteworthy that our microarray analysis shows that the FGF signaling pathway is activated by BBP treatment in R2d cells. It has been shown that overexpression of FGF increases the proportion of ER-positive cancer initiating cells induced by estradiol treatment [37]. Thus, our results support that the R2d cells possesses tumor-initiating characteristic which is enhanced in response to estrogenic stimuli.

Our results demonstrate that BBP stimulation is closely associated with enhanced tumorigenic characteristic of R2d cells because (1) gene expression profiling using a high-density microarray revealed significant activation of genes involved in cell proliferation, EMT, and angiogenesis, corroborating our earlier finding that estrogen promotes tumorigenicity of R2d cells through inducing Ki-67, EMA, MMPs, and VEGF [12], (2) the observed increase in CD44 and decrease of CD24 surface markers by BBP constitute a tumorigenic signature in breast cancer [11], [36], [38], (3) BBP indeed promotes invasiveness and angiogenesis of R2d cells in vitro and in vivo. We are aware that tumorigenicity is a complex process and multiple functions of cancer cells are involved. Our assays directly measure important cellular functions for tumor progression. Therefore, we refer these alterations as transforming phenotypes of R2d cells which can be enhanced by BBP treatment.

We would like to point out that tumor formation derived from MCF-7 is estrogen-dependent. Estrogen stimulation increases side population of R2d and MCF-7 cells [12], [13], [38]. Although MCF-7 cells express low level of CD44, BBP treatment significantly increases its CD44 expression (data not shown). Thus, both MCF-7 and R2d cells depend on estrogenic stimulation to form tumor, and respond to BBP with increased CD44 and decreased CD24. These results together suggest a general mechanism of exposure to BBP, and possibly other environmental endocrine disruptors, in breast cancer development and progression. We further interrogated the underlying gene network and identified that LEF-1 plays a pivotal role in the transforming activity of BBP in R2d cells.

LEF-1 is a known transcription factor involved in the Wnt/β-catenin signaling pathway [41]. The Wnt/β-catenin/LEF-1 pathway plays an important role in cancer stem cell biology [33], and is required for maintaining properties that regulate self-renewal and differentiation of cancer stem cells [42], [43]. When stimulated by β-catenin in the nucleus, LEF-1 complexes with the T cell factor (TCF) to activate gene expression through estrogen-responsive elements (EREs) of the targeted promoters [44], thus to re-program gene expression to enhance proliferation [45] and invasiveness [46]. LEF-1 is a known positive mediator of estrogen-induced mesenchymal transition in breast epithelial cells [47], [48]. Our current study shows that BPP induces LEF-1 expression to promote cell growth, invasion and migration in breast cancer. Our study also predicted the pathway in which LEF-1 is associated with endothelial nitric oxide synthase and the matrix metalloproteinase (MMP) family. This finding is supported by previous studies showing that LEF-1 increases the expression of nitric oxide synthase through prostaglandin-induced [49]. LEF-1 has also been shown to bind to the promoters of MMP-7/26 genes, leading to initiation of tumorigenesis [50], [51]. Therefore, our studies established that LEF-1 plays a critical role in the estrogenic effects of BPP in breast cancer cells.

We and others have shown that phthalate exposure results in pronounced cell transformation, both in vitro and in tumor xenograft models [30]. In the current study, we show that the BBP-LEF1 cascade observed in R2d cells can be recapitulated in the ER-positive breast cancer cell line MCF-7. Given that the R2d cells share certain phenotypes characterized as breast cancer initiating cells [12], our study conveys the important implication that environmental endocrine disruptors such as BBP may have a profound impact on breast cancer etiology by stimulating formation as well as progression of breast cancer. Our finding suggests that BBP treatment can induce tumor development derived from cancer stem-like cells. Studies are ongoing to test this hypothesis by measuring the tumor-initiating activity of R2d cells in response to BBP, and determine whether exposure to phthalates affects the responsiveness of R2d-derived breast tumors to chemotherapies.

In conclusion, we have shown that the cancer progression effect of BBP is due to its extensive impact on various biological functions, such as activation of proliferation, EMT, and angiogenesis via induction of LEF-1 expression. These findings contribute to our understanding of the tumorigenic potential of BBP and suggest possible strategies for preventing and treating breast cancer in humans.

## Materials and Methods

### Cell lines

The R2d cell line, which is comprised of human breast epithelial cancer stem cells, was obtained from Prof. CC Chang (Michigan State University, East Lansing, Michigan, USA) [12] and cultured in MSU-1 medium supplemented with 10% fetal bovine serum (Sigma, St. Louis, MO, USA) and 5% antibiotic antimycotic solution (100×) (Sigma). Dr Chang CC reported a tumorigenic cell line, M13SV1R2, which was developed from human breast epithelial cell type (Type 1 HBEC) with stem cell characteristics after successive immortalization by SV40 large T-antigen and transformation by X-ray irradiation [28]. If M13SV1R2 cell line cultured in growth factor/hormone-deprived medium (referred to as R2d cells), it lost the ability to develop tumor and became non-tumorigenic. The mammary cell line MCF-7 (ATCC) and HUVECs were grown in Dulbecco's modified Eagle's medium/F12 (Life Technologies, Grand Island, NY, USA) and EGM-2 medium, respectively, (Lonza Switzerland) under similar conditions. All cells were cultured at 37°C in an atmosphere of 5% CO_2_.

### BBP and the XTT assay

BBP, a phthalates ester, was purchased from Calbiochem (San Diego, CA, USA). To examine the effects of BBP on the growth of R2d cells, the cells were seeded and cultured for 24 h in a 96-well plate, followed by incubation in serum-free medium overnight, and then incubation in medium containing various concentrations of BBP for 24 h. The cells were analyzed at a wavelength of 490 nm and a reference wavelength of 650 nm for the XTT test solution (Sigma) with an ELISA reader (Multiskan EX; Labsystems, Vantaa, Finland). The XTT ratio was calculated as (absorbance of cells exposed to BBP)/(absorbance of cells not exposed to BBP).

### Flow cytometry

Cells were removed the culture medium and eluted twice in ice-cold PBS. After trypsinization, the single cells were obtained by straining through a 40 µM nylon mesh. The following antibodies were used for surface and intracellular staining: anti-CD44 (Santa Cruz Biotechnology, Santa Cruz, CA, USA), anti-CD24 (Santa Cruz Biotechnology), and anti-estrogen receptor (Santa Cruz Biotechnology), according to the manufacturer's recommendations. Cells were analyzed with an EPICS XL-MCL flow cytometer (Beckman Coulter, Miami, FL, USA).

### Total RNA extraction and quantitative real-time reverse transcription PCR

R2d cells were seeded and cultured for 24 h in a 10-cm dish, followed by incubation in serum-free medium overnight and then exposure to 1 µM BBP for 24 h. The cells were then washed with PBS at 4°C, and total RNA was isolated with Trizol solution (Invitrogen, Carlsbad, CA, USA). RNA (2 µg) was used to synthesize cDNA using a Reverse Transcription System kit (Promega, Madison, WI, USA). Samples were stored at −80°C until use. The candidate genes from the microarray analysis were confirmed with quantitative real-time PCR using the SYBR Green PCR Master Mix (Applied Biosystems, Foster City, CA, USA). Fluorescence was detected with the ABI-7500 system (Applied Biosystems). The PCR primers were as follows: CRIPTO forward: 5′-AA ATT AGA GGC CCC AGC ATT-3′, reverse: 5′-CAA AAG AAT GCC ATG TCT GC-3′; FGF forward: 5′-GGT GCA CTT TCT TCG GAT G-3′, reverse: 5′-ACG CCA TAC TAC AGG GGA TG-3′; PKA forward: 5′-CAC CAA GTA TCT CCC CAG A-3′, reverse: 5′-TAG CTG TGT TGC TGG GAC A-3′; EDNRA forward: 5′-TTG CCC ACA GCA GAC TAA AA-3′, reverse: 5′-GGT CAG AAA AGT CTG CAG CAA-3′; LMO1 forward: 5′-GTC TGT GTG TGA CCC CTC CT-3′, reverse: 5′-CCC CTA CCC TGC TCT GT-3′; VEGF-D forward: 5′-AGG AAG GAG ATT GGG TGA ATC-3′, reverse: 5′-GCA CCA AGG GGA AAA ATTA-3′; LEF-1 forward: 5′-CTT CTC CCC TCA TCC AAC TCT-3′, reverse: 5′-ACC AAA AGG CAA GCA GAG G-3′; PDGFR forward: 5′-GAA AGA TTG GGG CAA GTC TG-3′, reverse: 5′-GCA AAC AGG AAG TGC TCT GA-3′; PDGFRA forward: 5′-TGC TGT TAA TAT TTT CTG TTG TGC-3′, reverse: 5′-GAG TAC TGG TAA TAA ACC CAT GCAG-3′; ADCY forward: 5′-CAC GTC CTT TGG GAG ACT GT-3′, reverse: 5′-CCA TGG AGT GTG GAG AA ACC-3′; **TCF(LEF)** forward: 5′-CGG GGA AGA GCA GTA GAT GT-3′, reverse: 5′-TGT CAC AGT TTA AAA TGC ATC ATC-3′. The fold changes of the cycle threshold relative to the control value were calculated with the 2^−ΔΔ^Ct method for three independent experiments.

### cDNA microarray and data analysis

Total RNA (1 µg) from R2d cells was extracted with Trizol reagent and amplified with an Illumina Totalprep RNA Amplification kit (Ambion, Austin, TX, USA). Amplified cRNA samples were hybridized with streptavidin-Cy3 and scanned on the Illumina Beadstation GX. Samples (n = 2 in each group) were subjected to differential profiling using Illumina Beadstudio software, version 3.3.7., normalized with the Beadstudio cubic spline algorithm, and calculated with Beadstudio software according to the manufacturer's protocols. We selected genes whose expression changed more than ten-fold following BBP treatment compared with controls. To identify the molecular functions, canonical pathways involved in BBP treatment and the relationship between differentially expressed genes, we employed a manually curated proprietary database (MetaCore™, GeneGo, St. Joseph, MI), and the MetaCore pathway analysis software. The gene symbols of differentially expressed genes were uploaded into the database and enrichment analysis were performed according to the functional ontologies in MetaCore, including the GeneGo canonical pathway maps, GeneGo cellular processes, and the Gene Ontology (GO) cellular process and disease categories. Furthermore, we built a custom network by merging relative enriched functions ontologies and pathways.

### Wound healing and invasion assay

For the wound healing assay, the cells were seeded and cultured for 24 h in a 6-well plate. The cells were then scratched with a micropipette tip and washed to remove cellular debris, followed by treatment with BBP. A photograph of the wound distance was captured with an Olympus 1×71 microscope (Olympus, Tokyo, Japan) 24 h later, and the closure rate of the wound at three defined positions was calculated.

For the invasion assay, we used a Cell Invasion Assay kit (Chemicon, Temecula, CA, USA). Cells were seeded into the top chamber with 8-µm pore size inserts and treated with BBP in the upper chamber of a Falcon 48-well plate (BD Biosciences, San Jose, CA, USA). At the end of the treatment, invading cells were stained with crystal violet, and images were captured using a light microscope. Then, the cells were counted and analyzed in three independent fields.

### Tube formation assay

Tube formation was detected with the BioCoat™ Angiogenesis System (BD Biosciences) according to the manufacturer's protocol. The HUVEC (2×10^4^ cells) were seeded onto the Matrigel-precoated 96-well and added the medium that was obtained from the MCF-7 replacing medium with or without BBP. Angiogenesis was captured with a DM16000 B microscope (Leica, Bannockburn, IL, USA) and analyzed with MetaMorph software (Molecular Devices).

### Matrigel plug angiogenesis assay

To study the effects of BBP in the in vivo angiogenesis assay, phenol red–free Matrigel (BD Biosciences) was used in female nude mice (BALB/cAnN.Cg-Foxn1nu/CrlNarl, 4–6 weeks old), which were obtained from the National Laboratory Animal Center (Taipei, Taiwan). The experiment was conducted according to the protocol approved by the Kaohsiung Medical University Hospital Institutional Animal Care and Use Committee (IACUC Approval No: 980187). Mice were randomly divided into two treatment groups. Cell suspensions with or without BBP were mixed with 0.4 mL matrigel and injected into the flanks of each mouse. Fifteen days after implantation, blood vessel formation was stained, and the hemoglobin value was determined with Drabkin's reagent kit (Sigma-Aldrich).

### Transfection of RNA oligonucleotides and immunoblotting

siRNA transfection and immunoblotting were performed as described [9]. The following siRNAs were used: control siRNA (sense: 5′-GAU CAU ACG UGC GAU CAG A-3′, antisense: 5′-UCU GAU CGC ACG UAU GAU C-3′); LEF-1 siRNA-1 (GenBank SASI_00151593; sense: 5′-GAA ACU ACA GGA AUC UGCA-3′, antisense: 5′-UGC AGA UUC CUG UAG UUUC-3′); LEF-1 siRNA-2 (GenBank SASI_00349169; sense: 5′-CAU CAG AUG UCA ACU CCA A-3′, antisense: 5′-UUG GAG UUG ACA UCU GAUG-3′).The following antibodies used for immunoblotting were purchased: anti-LEF-1(Origene Technologies, Inc., Rockville, MD) and anti-β-actin (Sigma)
